# Association between obesity and mortality in the Costa Rican elderly: a cohort study

**DOI:** 10.1186/s12889-022-13381-9

**Published:** 2022-05-18

**Authors:** Carolina Santamaría-Ulloa, Anne Chinnock, Melina Montero-López

**Affiliations:** 1 Health Research Institute, University of Costa Rica, San Pedro, Costa Rica; 2 Human Nutrition Department, University of Costa Rica, San Pedro, Costa Rica; 3 Health Research Institute, University of Costa Rica, San Pedro, Costa Rica

**Keywords:** Obesity, Mortality, Elderly population, Chronic conditions

## Abstract

**Background:**

Costa Rica, along with other Latin American countries, is undergoing population aging, with an increase in the prevalence of chronic conditions, many of them resulting from a growing prevalence of obesity. As a result of the demographic and epidemiological transitions, the causes of morbidity and mortality have changed from communicable to non-communicable diseases. An increase in overweight and obesity is hypothesized to be related to premature mortality. This study assesses the association between obesity and both all-cause and cardiovascular mortality in a Costa Rican elderly population.

**Methods:**

This is a secondary data analysis of the Costa Rican Longevity and Healthy Aging Study (CRELES, for its Spanish acronym), a longitudinal nationally representative cohort study of health and life-course experiences of the Costa Rican elderly. A baseline (*n* = 2827) and two subsequent 2-year follow-up interviews were conducted. Data analyses include descriptive statistics and survival models. Cox and Gompertz distributions were used to model general mortality and cardiovascular mortality as a function of obesity and controlling for confounders. Anthropometric measures used were Body Mass Index (BMI) and Waist Circumference (WC).

**Results:**

Each one-unit increment in BMI was significantly associated to a 3,1% (*p <* 0.001) and 2,6% (*p* = 0.021) increment in general and cardiovascular mortality respectively. Each one-unit increment in WC was significantly associated with a 0,8% (*p* = 0.006) increment in general mortality, whereas WC was not significantly associated with cardiovascular mortality.

**Conclusions:**

Obesity is associated with mortality in the Costa Rican elderly. This association is strongest between obesity and all-cause mortality. As general obesity increases, all-cause and cardiovascular mortality also increase in this population. Similarly, as central obesity increases, all-cause mortality increases. Policies aimed at preventing obesity and chronic conditions are warranted for a better survival in this population.

## Background

Latin American developing countries are experiencing the process of demographic aging at a very rapid pace. The time it will take for any Latin American country to have an ageing population is less than two fifths of the time it took for the United States to reach that composition. In Costa Rica, by 2021, 14% of the total population was aged 60 years or older [1].

Aging populations experience an increase in the prevalence of chronic conditions, many of them resulting from an increasing prevalence of obesity. As a result of the demographic and epidemiological transitions, the causes of morbidity and mortality have changed from communicable to non-communicable diseases in Costa Rica [2]. In practically all populations, the adoption of diets with a higher fat content and reduced physical activity, have accompanied the benefits of modernization. These changes in diet and physical activity levels, combined with longevity, are the basis for an increasing prevalence of obesity.

Overweight and obesity are understood as the excess of body fat, which results of the imbalance between food intake and energy expenditure [3]. Obesity levels in Latin America and the Caribbean are as high, or even higher than obesity levels in the United States. In adulthood, obesity is associated with an increased prevalence of chronic diseases, and higher rates of all-cause and cardiovascular mortality [4–6]. A meta-analysis of 239 different studies from 32 countries in Asia, Australia, New Zealand, Europe and North America found that in different regions, except for South Asia, there was a strong and positive relationship between overweight and all-cause mortality, especially in the case of men [4].

Furthermore, obesity has been found to contribute to the risk of cardiovascular mortality. Metabolic complications of obesity can potentiate each other. Type 2 diabetes and high blood pressure, for example, may lead to chronic kidney disease, one of the possible causes of early disability [7]. As described in previous studies, there is a strong positive relationship between obesity and mortality due to cardiovascular diseases [8–10]. Central obesity, measured as waist circumference or as its relationship with hip and height, has shown to be effective when studying cardiovascular risk [11].

Although disability is higher among obese elderly, an obesity paradox in the oldest of the elderly has been described in the literature, especially after the age of 80. This paradox refers to an attenuation of the negative effect that obesity has on mortality as age increases [12, 13]. It has been previously described that because of the loss of weight that is associated with aging, a degree of obesity can be a protective factor against mortality [14–16]. There is no consensus about the detrimental effect of obesity on mortality in the elderly population [17]. Nonetheless, premature death is expected to be the result of obesity at least in the younger elderly [16], before the age of 80.

Most of the studies on the association between obesity and mortality have been conducted in developed countries. In Latin America most of research has been conducted on infant mortality and on transmissible diseases. Nonetheless, Suemoto (2015) conducted a study in 7 Latin-American developing countries, which did not include Costa Rica, and found that participants with an obese-related condition such as type 2 diabetes had an increased mortality risk [18]. Studying the same Costa Rican elderly cohort that is used for this study, Santamaría (2020) found an association between premature mortality and type 2 diabetes. They also described geographical and gender inequalities in the prevalence of diabetes in Costa Rica [19]. There is a gap of evidence on the relationship between obesity and mortality in the context of Latin American elderly.

There is also not enough evidence that risk factors traditionally associated with mortality in adulthood in developed countries are the same risk factors associated with mortality in the elderly in developing countries. Until recently, no representative elderly population studies were available in Latin America. This data limitation has been overcome in Costa Rica with the CRELES study, a nationally representative cohort study of the elderly population with baseline data collected between 2004 and 2006 and whose participants’ survival has been followed up ever since.

The aim of this study is to assess the association between obesity and both all-cause and cardiovascular mortality in a Costa Rican elderly cohort. This study provides evidence on the relationship between obesity and mortality among the elderly in developing countries for which population data is not widely available. This research contributes to the field of public health by providing decision makers with recommendations to attain healthier and longer lives for the elderly population.

## Methods

This is a secondary data analysis of the Costa Rican Longevity and Healthy Aging Study (CRELES, for its Spanish acronym), a nationally representative cohort study of health and life-course experiences of the Costa Rican elderly.

A baseline (*n =* 2827) and two subsequent 2-year follow-up interviews were conducted. Data collection occurred between 2004 and 2006 for the baseline, between 2006 and 2007 for the second wave, and between 2008 and 2009 for the third wave. Loss to follow-up between baseline and wave 2 was 7%, and between waves 2 and 3 it was 9% of the baseline sample.

### Sample design and selection

The study sample was probabilistic, two-staged and stratified. In the first stage, individuals 55 years or over, were randomly selected from the Costa Rican population census of 2000. In the second stage, the individuals were classified according to residence as defined by the Ministry of Health’s geographical areas and the final sample contained 2827 individuals [15].

Data collection was conducted via a structured interview, anthropometric measures and blood and urine samples [20], all carried out in the homes of the selected subjects. During the first interview, all subjects signed an informant consent form, responded to the questionnaire and anthropometric measures were taken. During the second visit on the following day, blood and urine samples were collected [20].

Respondent’s vital status was assessed during the three waves of CRELES, and it was also tracked by linking the CRELES dataset with the National Death Registry up to 31 October 2017. More details on this survey have been previously published [19–22].

### Variables

Waist circumference (WC) and body mass index (BMI) were used as indicators of central and general obesity respectively. Body mass index was estimated as the individual’s weight divided by his squared height. Both anthropometric measures were used as continuous variables in regression models. Descriptive data for these anthropometric measures is presented as categorical variables. According to BMI, individuals were classified as underweight (< 18.5 kg/m2), normal weight (18.5–24.9), overweight (25.0–29.9), or obese (≥30.0 kg/m2) [23]. Waist circumference categories for men were normal (< 94 cm), with an increased risk of metabolic complications (94–101), and substantially increased risk of metabolic complications (≥102 cm). Waist circumference categories for women were normal (< 80 cm), with increased risk of metabolic complications (80–87), and substantially increased risk of metabolic complications (≥88 cm) [23].

Body weight, height and waist circumference were measured during fieldwork. A scale and a stadiometer were placed in a firm and flat place in each participant’s house. Participant subjects were asked to stand on the scale to obtain the weight measurements. They were also asked to stand next to the scale, controlling their anatomical points, in order to measure height. Waist circumference measurements were taken with a tape measure. Participants were asked to stand in, and to indicate the level at which their belly button was. Waist circumference was measured around the abdomen at a level midway between the lowest rib and the upper hip bone. In participants with large deformations in the spine or with stability problems, waist circumference was not measured.

In addition to BMI and WC, analyses controlled for sex, education, smoking, alcohol consumption, having a fracture in the last 6 months, and the following morbidity conditions: hypertension, hypercholesterolemia, diabetes mellitus, cancer, heart attack, ischemic heart disease (without infarction), and stroke.

Education was classified into incomplete primary and complete primary or higher. Smoking behavior refers to 100 or more cigarettes or cigars during participants’ lives. Categories were defined as never smoked, former smoker, passive smoker, and current active smoker. Individuals living with a smoking partner were classified as passive smokers, when they were not active smokers themselves. Alcohol consumption refers to alcoholic beverages ever consumed during individuals’ lives. Categories were defined as never drank alcohol, former drinker, occasional drinker, and current drinker. Smoking and alcohol consumption categories used in this study are similar to those used by Koawall et al. [24] and Ye et al. [25] respectively.

### Survival analysis

Survival analysis is a collection of statistical procedures for data analysis for which the outcome variable is the time elapsed until an event occurs [26]. This is an appropriate statistical technique when working with longitudinal studies where the subjects’ follow-up time is available and a binary variable indicates whether or not the observed variable is censored [27].

The data was set as survival time. Follow-up time started at the date each individual was 60 and ended the date the subject died or the observation time ended on October 31, 2017. Although interviews were not necessarily conducted at the age of 60 for each participant during the first wave, date at the age of 60 was taken as the starting observation point because all the participant subjects were alive at that time. Mortality rates were computed as the ratio of deaths to the exact count of person-years.

Parametric survival regression models were used to estimate the association between mortality and obesity. Cox and Gompertz distributions were used to model mortality. Proportional risks Cox models, widely used in health sciences, were used under the assumption that mortality risks are proportional to time. Models with a Gompertz distribution for the baseline hazard were used to model mortality [28]. They are similar to Cox models, with the difference that the baseline risk is specified as a Gompertz shape parameter [27]. Costa Rican mortality rates have shown to follow a Gompertz function, especially after the age of 45 [29].

For these survival regression models, mortality was measured both as general (all-cause) and as cardiovascular. Obesity was measured both as central (WC) and as general (BMI). Control variables were sex, education, smoking, alcohol consumption, having a fracture in the last 6 months, and the following morbidity conditions: hypertension, hypercholesterolemia, diabetes mellitus, cancer, myocardial infarction, ischemic heart disease (no infarction), and stroke.

Because of the short time elapsed between waves (21 months between first and second wave, and 24 months between second and third wave), anthropometric measures showed no relevant change. BMI, WC and all of the covariates were therefore used as baseline measures and no time-dependent variables were considered in the survival analyses conducted.

## Results

A general description of this cohort is presented in Table 1. There is a higher proportion of female elderly population, aged 60 to 69, with an almost equal distribution regarding educational status. Less than half (43%) of this elderly population has a normal BMI, whereas 51% of men and 13% of women have a normal WC.

**Table 1:** Percentage distributions of Costa Rican elderly according to sociodemographic characteristics, Body Mass Index and Waist Circumference

Characteristics	Percentage
Total	100
**Sex**	
Male	47.48
Female	52.52
**Age**	
60-69 yrs.	53.26
70-79 yrs.	31.74
80+	15
**Education**	
Incomplete primary	50.69
Complete primary and more	49.31
**Body Mass Index (BMI)**^**a**^	
Underweight (<= 23 kg/m2)	20.62
Normal (23.1-27.9 kg/m2)	43.29
Overweight (28-31.9 kg/m2)	23.21
Obese (32+ kg/m2)	12.88
**Waist circumference**	
**Male**	
Normal (< 94 cm)	51.49
Increased (94-101 cm)	26.45
Substantially increased (102+ cm)	22.07
**Female**	
Normal (< 80 cm)	13.47
Increased (80-87 cm)	20.2
Substantially increased (88+ cm)	66.33

^a^ World Health Organization (OMS), 2002

A description of risk factors is presented in Table 2. About a third of elderly are former smokers or alcohol drinkers, whereas 10% are current smokers and 3% are current alcohol drinkers. Hypertension and hypercholesterolemia are the most prevalent conditions on this elderly population (48 and 40% respectively), 5% have had a myocardial infarction and 12% have ischemic heart disease.

**Table 2:** Percentage distributions of Costa Rican elderly according to risk factors and health condition

Characteristics	Percentage
Total	100
**Risk factors**	
**Smoking**	
Never	35.55
Former smoker	33.09
Passive smoker	21.38
Current smoker	9.98
**Alcohol consumption**	
Never	35.78
Former drinker	31.27
Occasional drinker	30.26
Current drinker	2.68
**Health condition**	
**Hypertension**	48.35
**Cholesterol**	39.56
**Diabetes**	20.85
**Cancer**	5.85
**Myocardial infarction**	4.58
**Ischemic heart disease (no infarction)**	12.11
**Stroke**	3.83
**Fracture in the last 6 months**	12.30

**Fig. 1:**
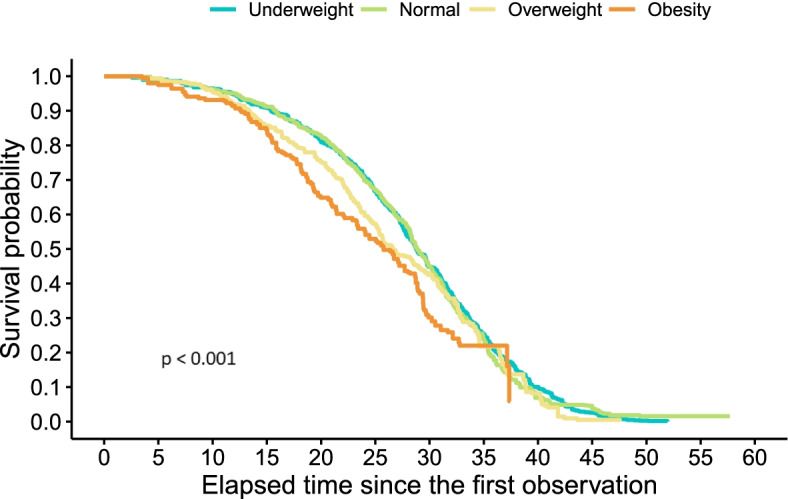
Survival to general mortality by BMI

**Fig. 2:**
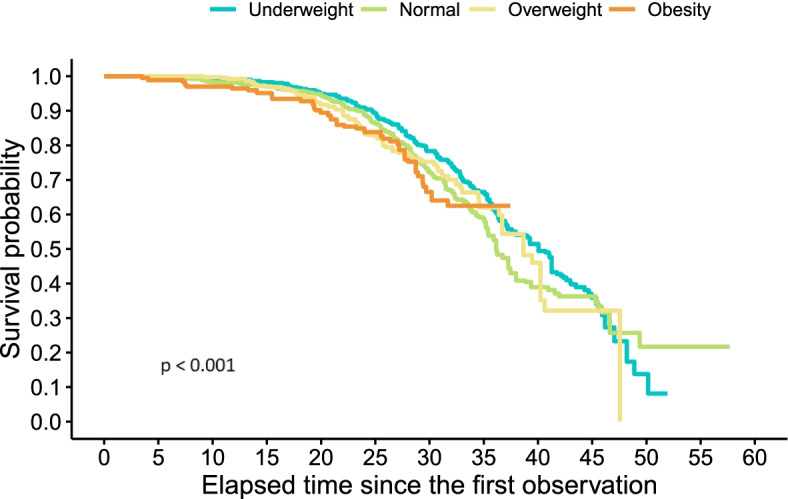
Survival to cardiovascular mortality by BMI

Cox survival models on general and cardiovascular mortality by BMI are presented in Fig. 1 and Fig. 2 respectively. From the age of 60 (elapsed time since first observation = 0) up to the age of 95 (elapased time = 35), the obese population has the lowest probability of surviving death due to any cause (Fig. 1).

Similarly, from the age of 60 up to the age 90, the obese elderly have the lowest probability of surviving death due to cardiovascular disease (Fig. 2). Death associated with obesity seems to occur by the age of 90 or 95 in this elderly cohort. After this age threshold, survival probabilities by BMI intersect with each other.

Gompertz regression results for general mortality are presented in Table 3. Each one-unit increment in WC was significantly associated to a 0,8% (*p* < 0.001) increase in general mortality. Along the same lines each one-unit increment in BMI was significantly associated to a 3,1% (*p <* 0.001) increase in all-cause mortality. The odds of death are significantly higher for males and for active smokers. Diabetes and myocardial infarction are significantly associated with all-cause mortality. Stroke is associated with all-cause mortality when obesity is measured by WC but not when it is measured by BMI.

**Table 3:** All-cause mortality: Odds ratios from Gompertz parametric models with waist circumference or body mass index as main predictor

Model with waist circumference				Model with Body Mass Index			
**Variable**	**OR**	**Associated probability**		**Variable**	**OR**	**Associated probability**	
Waist circumference	1.008	*p <* 0.001	***	Body Mass Index (BMI)	1.031	*p <* 0.001	***
Male	1.346	*p <* 0.001	***	Male	1.378	*p <* 0.001	***
Complete primary	1.119	0.046	**	Complete primary	1.095	0.090	
Former smoker^a^	0.997	0.512		Former smoker^a^	1.015	0.429	
Passive smoker	0.909	0.842		Passive smoker	0.887	0.892	
Current smoker	1.704	*p <* 0.001	***	Current smoker	1.808	*p <* 0.001	***
Former drinker^b^	0.881	0.905		Former drinker^b^	0.872	0.917	
Occasional drinker	0.803	0.988		Occasional drinker	0.788	0.991	
Current drinker	0.899	0.691		Current drinker	0.862	0.748	
Hypertension	1.090	0.103		Hypertension	1.089	0.114	
Cholesterol	0.975	0.638		Cholesterol	0.959	0.716	
Diabetes	1.444	*p <* 0.001	***	Diabetes	1.423	*p <* 0.001	***
Cancer	1.082	0.257		Cancer	1.043	0.371	
Myocardial infarction	1.511	*p <* 0.001	***	Myocardial infarction	1.443	0.002	**
Ischemic heart disease (no infarction)	1.087	0.182		Ischemic heart disease (no infarction)	1.111	0.127	
Stroke	1.332	0.017	**	Stroke	1.147	0.186	
Fracture in the last 6 months	0.858	0.960		Fracture in the last 6 months	0.831	0.978	

Significance levels: ***p < 0.01; ** *p* < 0.05; * *p* < 0.10

People who have never smoked are the reference category^a^

People who have never drunk are the reference category^b^

Regression results for cardiovascular mortality are presented in the Table 4. Each one-unit increment in BMI was significantly associated to a 2.6% (*p* = 0.021) increase in cardiovascular mortality. The odds of cardiovascular death are significantly higher for males and for active smokers. Hypertension, diabetes, myocardial infarction, and stroke are significantly associated with cardiovascular mortality.

**Table 4:** Cardiovascular mortality: Odds ratios from Gompertz parametric models with waist circumference and body mass index as main predictor

Model with waist circumference				Model with Body Mass Index			
Variable	OR	Associated probability		Variable	OR	Associated probability	
Waist circumference	1.005	0.134		Body Mass Index (BMI)	1.026	0.021	**
Male	1.331	0.032	**	Male	1.414	0.016	**
Complete primary	1.086	0.243		Complete primary	0.967	0.604	
Former smoker^a^	1.033	0.413		Former smoker^a^	0.989	0.527	
Passive smoker	0.924	0.684		Passive smoker	0.898	0.738	
Current smoker	2.147	*p <* 0.001	***	Current smoker	2.334	*p <* 0.001	***
Former drinker^b^	0.983	0.538		Former drinkerb	0.919	0.685	
Occasional drinker	0.857	0.813		Occasional drinker	0.855	0.809	
Current drinker	1.153	0.348		Current drinker	1.174	0.334	
Hypertension	2.002	*p <* 0.001	***	Hypertension	2129	*p <* 0.001	***
Cholesterol	1.072	0.277		Cholesterol	1.057	0.325	
Diabetes	1.662	*p <* 0.001	***	Diabetes	1.600	*p <* 0.001	***
Cancer	0.841	0.767		Cancer	0.852	0.739	
Myocardial infarction	1.719	0.002	**	Myocardial infarction	1.592	0.012	**
Ischemic heart disease (no infarction)	1.202	0.107		Ischemic heart disease (no infarction)	1.277	0.051	.
Stroke	1.813	0.001	***	Stroke	1.27	0.160	
Fracture in the last 6 months	0.860	0.843		Fracture in the last 6 months	0.875	0.803	

Significance levels: ***p < 0.01; ** *p* < 0.05; * *p* < 0.10

People who have never smoked are the reference category^a^

People who have never drunk are the reference category^b^

Results from Cox proportional hazards are similar to those using Gompertz models both for all-cause mortality and for cardiovascular mortality. Each one-unit increment in WC was significantly associated to a 0,8% (*p* = 0.002) increase in general mortality. Each one-unit increment in BMI was significantly associated to a 2,9% (*p <* 0.001) increase in all-cause mortality (Table 5).

**Table 5:** All-cause mortality: Odds ratios from Cox models with waist circumference or body mass index as main predictor

Model with waist circumference				Model with Body Mass Index			
Variable	OR	Associated probability		Variable	OR	Associated probability	
Waist circumference	1.008	0.002	***	Body Mass Index (BMI)	1.029	*p <* 0.001	***
Male	1.357	*p <* 0.001	***	Male	1.388	*p <* 0.001	***
Complete primary	1.112	0.111		Complete primary	1.089	0.211	
Former smoker^a^	0.994	0.949		Former smoker^a^	1.013	0.875	
Passive smoker	0.927	0.423		Passive smoker	0.909	0.324	
Current smoker	1.676	*p <* 0.001	***	Current smoker	1.781	*p <* 0.001	***
Former drinker^b^	0.875	0.162		Former drinkerb	0.864	0.138	
Occasional drinker	0.792	0.016	**	Occasional drinker	0.777	0.010	**
Current drinker	0.905	0.605		Current drinker	0.862	0.503	
Hypertension	1.072	0.309		Hypertension	1.07	0.331	
Cholesterol	0.966	0.622		Cholesterol	0.951	0.482	
Diabetes	1.431	*p <* 0.001	***	Diabetes	1.409	*p <* 0.001	***
Cancer	1.075	0.548		Cancer	1.037	0.777	
Myocardial infarction	1.496	*p <* 0.001	***	Myocardial infarction	1.428	0.005	***
Ischemic heart disease (no infarction)	1.083	0.385		Ischemic heart disease (no infarction)	1.109	0.265	
Stroke	1.328	0.035	**	Stroke	1.143	0.385	
Fracture in the last 6 months	0.893	0.192		Fracture in the last 6 months	0.866	0.114	

Significance levels: ***p<0.01; ** *p*<0.05; * *p*<0.10

People who have never smoked are the reference category^a^

People who have never drunk are the reference category^b^

According to the Cox regression models for cardiovascular mortality (Table 6), a significant difference in risk of death was observed between men and women (*p* = 0.029) which indicates that men have a 42% greater risk of death as compared to women. Hypertension was found to have a significant effect on the probability of death and individuals with this condition have double the risk of death compared to those without this health condition. A similar situation was found for individuals with diabetes and for individuals who had suffered myocardial infarction; in these cases, the risk of death was increased by 55.8 and 56.2% respectively. Smoking also had a significant effect on the risk of death, whereby current smokers had more than double the risk as compared to those who had never smoked.

**Table 6:** Cardiovascular mortality: Odds ratios from Cox models with waist circumference and body mass index as main predictor

Model with waist circumference				Model with Body Mass Index			
**Variable**	**OR**	**Associated probability**		**Variable**	**OR**	**Associated probability**	
Waist circumference	1.004	0.345		Body Mass Index (BMI)	1.023	0.065	*
Male	1.343	0.056	*	Male	1.422	0.029	**
Complete primary	0.089	0.471		Complete primary	0.973	0.830	
Former smoker^a^	0.032	0.933		Former smoker^a^	0.987	0.933	
Passive smoker	0.939	0.704		Passive smoker	0.916	0.604	
Current smoker	2.093	*p <* 0.001	***	Current smoker	2.268	*p <* 0.001	***
Former drinker^b^	0.977	0.891		Former drinker^b^	0.915	0.609	
Occasional drinker	0.847	0.340		Occasional drinker	0.847	0.350	
Current drinker	1.160	0.683		Current drinker	1.170	0.676	
Hypertension	1.929	*p <* 0.001	***	Hypertension	2.042	*p <* 0.001	***
Cholesterol	1.074	0.547		Cholesterol	1.059	0.638	
Diabetes	1.623	*p <* 0.001	***	Diabetes	1.558	0.001	***
Cancer	0.842	0.467		Cancer	0.85	0.516	
Myocardial infarction	1.687	0.006	***	Myocardial infarction	1.562	0.029	**
Ischemic heart disease (no infarction)	1.202	0.215		Ischemic heart disease (no infarction)	1.284	0.094	
Stroke	1.812	0.002	***	Stroke	1.268	0.324	
Fracture in the last 6 months	0.906	0.507		Fracture in the last 6 months	0.921	0.596	

Significance levels: ***p<0.01; ** *p*<0.05; * *p*<0.10

People who have never smoked are the reference category^a^

People who have never drunk are the reference category^b^

The Cox regression model incorporating WC found no significant difference in the probability of death from cardiovascular causes between men and women. Among individuals with hypertension, diabetes, myocardial infarction or stroke, the risk of death from cardiovascular causes was significantly greater, especially for individuals with hypertension, whose risk doubled the risk as compared to those without this condition. Current smokers were also found to have an increased risk of death from cardiovascular causes.

## Discussion

Results from this study show that general and central obesity, as measured by BMI and WC respectively, are associated with all-cause and with cardiovascular mortality in the elderly. In terms of magnitude as measured by each one-unit increment, the greatest association was found between BMI and all-cause mortality, followed by the association between BMI and cardiovascular mortality. General obesity seems to be a good predictor of mortality in the elderly. BMI is a relatively easy to obtain biomarker, that estimates the general level of body fat. It is widely used; it has shown to be associated with cardiovascular risk [30] and correlates positively with other biomarkers such as waist circumference or waist-to-hip ratio [31]. Although BMI does not adequately differentiate relevant elements from of body weight and it can be better complemented with measures such as WC [32]. It has shown to be associated with both all-cause and cardiovascular mortality in this elderly population.

WC was found to be significantly associated with all-cause mortality but not with cardiovascular mortality. Cardiovascular disease is usually a comorbid condition, which is not always reported as the main cause of death. This may be the reason why no significant association was found between WC and cardiovascular mortality. Waist circumference is a useful measure because of its direct relation with central or abdominal adiposity [17], which is associated with a number of metabolic anomalies [31]. It is associated with visceral and total fat assessed by computerized tomography [33, 34]. Although WC as a biomarker is not difficult to obtain, it is not as widely used as BMI on population studies. Using BMI in the absence of measures such as WC could be misleading [5]. The use of BMI in this study is advantageous because it allows for comparability with other studies, whereas its use combined with WC is more appropriate as it allows for a finer measure of abdominal obesity.

Similar to what has been found for the same cohort in other studies [15], when using BMI as a categorical variable, there seems to be a threshold around the age of 90, which survival probabilities are not associated with obesity anymore. Some studies suggest that as we grow older, the association between mortality and obesity becomes weaker [35]. Whereas other studies [36, 37] have described that the so-called obesity paradox may actually be the result of not studying central, but general adiposity in the elderly.

The association between obesity and mortality has been explored by other studies. A10-year follow-up study found that, after adjusting for other confounding variables, the mortality risk was 2.46 higher for elderly with dynapenic abdominal obesity [38]. A study conducted in Brazil and United Kingdom also identified that, general and central obesity, were associated with higher all-cause and cardiovascular mortality in the elderly [5].

Over two thirds of all-cause deaths that have been attributed worldwide to overweight and obesity occur specifically through cardiovascular disease [39]. Cardiovascular disease in the elderly is associated with biological mechanisms of nutritional depletion, systemic inflammation and physical inactivity, which lead to muscle mass decline processes and to mortality [40, 41].

As a society, obesity represents high costs that are not only financial, but physical an emotional [40]. Because of being a chronic condition, obesity represents an additional cost for healthcare providers and for the individual population, with a strong correlation between high BMI and medical expenditure in the United States of America [42].

Obesity is a common risk factor for a number of chronic diseases associated with an increased mortality, such as hypertension and diabetes. Elderly individuals with hypertension, for example, have twice the risk of death as compared to those who do not have this condition [43]. The fact that obesity is itself a risk factor for hypertension emphasizes the importance of policies for obesity prevention in the general population.

This study also found that sex was a significant predictor of mortality. Although in the general elderly population mortality in men is higher than in women, many of the extra years lived by women are spent in poor health [44]. Studies conducted in diabetic populations have found that woman with diabetes have a higher risk of mortality than men with the same condition [45]. Furthermore, multiple studies have also reported that the probability of having cardiovascular diseases is usually greater in men than in women throughout the life cycle; however, as age advances, it is women who begin to have an increased risk of cardiovascular diseases [46].

Smoking was significantly associated with mortality in this elderly cohort. There is a strong relationship between smoking and multiple diseases, such as acute coronary events, stroke, and cardiovascular disease. Previous studies have reached similar results, and have concluded that the risk of suffering from some cardiovascular disease can be up to double in smokers. There is also a clear gradient effect that is associated with the fact that quitting smoking reduces the risk of cardiovascular disease [47].

This study found that comorbidities that are significantly associated with mortality are similar to findings of other studies regarding the association between cardiovascular mortality and conditions such as hypertension [48] and stroke [49].

The increasing number of overweight and obese individuals has made obesity a challenge for the Costa Rican healthcare system because, as this study has shown, it predicts an increased mortality in the elderly population. Planning actions to reduce obesity, not only benefits the population’s quality of life, but also protects the sustainability of the healthcare system.

Information bias is a limitation of this study because of the use of self-reports for comorbid conditions. Selection bias is also a limitation because survey data relies on the population that survived at least to the age of 60 in 2005, when the baseline survey was conducted. Also, another limitation of this study is the impossibility of studying individual changes in obesity over time because of the short time-span between waves and the resultant small changes in nutritional status as indicated by the anthropometric measurements in this elderly population. Another limitation related specifically to cardiovascular mortality is that this study uses the immediate cause of death (the final disease or condition resulting in death) rather than the multiple underlying causes of death (conditions leading to the event listed as the immediate cause of death) that are captured in each individual death report. Given the fact that cardiovascular disease is usually a comorbid condition, this would have the effect of underestimating the effect of overweight and obesity on mortality in this population.

Despite these limitations, this study presented several strengths. Similar to what has been shown in other studies, the use of Waist Circumference besides the use of Body Mass Index, allowed for the identification of people with central obesity at a higher risk of all-cause mortality. Policy making is an implication expected from the evidence presented by this cohort study. As mentioned by Livingston [50], we must first understand the underlying factors of population obesity to be able to act upon reversing this worldwide trend. In order to really impact the population, social determinants of health need to be acknowledged in health policies to reduce obesity.

Policies aimed at preventing obesity and chronic conditions are warranted for a better survival in this population. Improving lifestyles and reducing obesity in the general population are needed to attain healthier and longer lives for the elderly. Also, public policies should have a gender component that takes into account special needs for both the male and female populations. Future lines of research are advised to be conducted on how individual changes in nutritional status over time can have an impact on mortality in the elderly population.

## Conclusions

Obesity is associated with mortality in the Costa Rican elderly. This association is strongest between obesity and all-cause mortality. As general obesity increases, all-cause and cardiovascular mortality also increase in this population. Similarly, as central obesity increases, all-cause mortality increases.

Reducing the prevalence of overweight and obesity along the lifecycle is certainly required as a public health policy in order to obtain life expectancy gains. This is especially true because the longer is the duration of overweight and obesity along the lifecycle of an individual, the greater is his risk of mortality [51–53].

## Data Availability

Data and materials from the CRELES study are available at https://ccp.ucr.ac.cr/proyectos/creles/presentacion

## Abbreviations

BMI: Body Mass Index
CRELES: Costa Rica Longevity and Healthy Aging Study, for its Spanish acronym
WC: Waist Circumference
